# Recurrent Retrorectal Teratoma

**DOI:** 10.1155/2014/491605

**Published:** 2014-03-23

**Authors:** P. Geoff Vana, Sherri Yong, Dana Hayden, Theodore Saclarides, Michelle Slogoff, William Boblick, Joshua Eberhardt

**Affiliations:** Department of Surgery, Loyola University Medical Center, 2160 South First Avenue, Maywood, IL 60153, USA

## Abstract

Retrorectal tumors are a rare group of neoplasms that occur most commonly in the neonatal and infant population. They vary in presentation, but teratomas are the most common and often present as a protruding mass from the sacrococcygeal region. Immediate surgical resection is indicated when found and coccygectomy is performed to prevent recurrence. When teratomas recur, the patients most often have vague symptoms and the tumors usually have malignant transformation. Here, we present the case of a young woman who underwent surgical resection of a sacrococcygeal teratoma at 3 days of age where the coccyx was not removed. She presented at 31 years of age with lower extremity paresthesias and radiography revealed a cystic mass extending from the sacrum. After resection, pathology revealed a recurrent teratoma with nests of adenocarcinoma.

## 1. Introduction

Retrorectal neoplasms refer to a rare and heterogeneous group of tumors that occur in what is known anatomically as the retrorectal space. This is a potential space defined superiorly by the rectal peritoneal reflection, anteriorly by the rectum, posteriorly by the sacrum, and inferiorly by the rectosacral fascia. Although the retrorectal space does not technically include the more inferiorly located supralevator and deep postanal spaces, lesions occurring within these areas are still generally referred to as retrorectal or presacral neoplasms.

As mentioned, retrorectal neoplasms are rare and occur more commonly in the neonatal population with a female : male ratio of 3-4 : 1 [1, 2]. They have a reported incidence as low as 1 in 40,000–60,000 hospital admissions at tertiary centers [3–5]. A wide variety of pathologic entities may develop in the retrorectal space owing to its complicated embryologic development and several classification systems have been proposed to describe them. The most commonly used taxonomy puts the lesions into the following categories: congenital, inflammatory, neurogenic, osseous, and miscellaneous [6, 7].

Of the congenital lesions, teratomas usually are found in the sacrococcygeal region and they continue to be the most common tumor found in newborns where 80% are found in the first six months of life [6, 7]. They are often found on initial physical exam as a large mass protruding from the sacrococcygeal area. The neonatal form is commonly benign, but those found in the adult population can possess malignant transformation potential if left untreated [8–10]. In these patients, the standard of care is immediate surgical excision taking care to define anatomic landmarks preoperatively and avoid nerve injury as bowel and bladder incontinence has been a result of resection of sacral nerve roots proximal to S3 [4, 11]. Controversy surrounds the necessity of coccygectomy during resection of presacral masses [3, 12–14]. Since teratomas arise from embryonic totipotent cells that theoretically arise from Henson's node and migrate caudally to rest in the coccyx, many advocate for coccygectomy when a sacrococcygeal teratoma is diagnosed, whether at birth or in an adult [6, 7, 9, 14, 15]. Due to this rest of cells, recurrence can be as high as 37% if coccygectomy is not performed [6, 14, 16]. As demonstrated by this case, when recurrence develops in a patient who previously had infantile sacrococcygeal teratoma, the recurrence can happen decades later and the presenting symptoms can be vague with insidious onset.

## 2. Case

**Figure 1:**
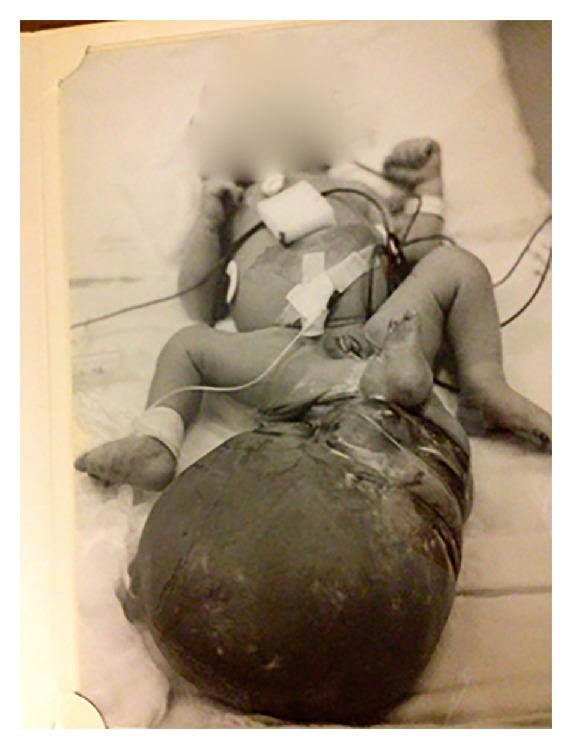
Original photograph of patient with congenital sacrococcygeal teratoma at 3 days of age before primary surgery.

**Figure 2:**
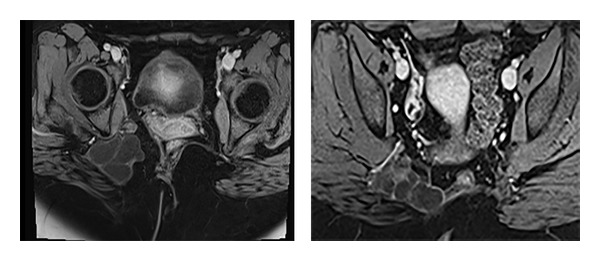
MRI pelvis with recurrent teratoma extending from the retrorectal space and precoccygeal area across midline to the region of right piriformis and deep to right gluteus muscles.

A 31-year-old obese female presented with a 6-month history of intermittent pelvic pain along with occasional right lower extremity paresthesias. Her only significant medical history was that at birth she was found to have a congenital sacrococcygeal teratoma (Figure 1). She underwent surgery for this at birth and subsequently developed normally without deficit. Now, at 31 years of age, she presented with the above symptoms. Physical exam revealed a well-healed surgical scar on her right buttock. She had no motor deficits and no abnormality on rectal or vaginal exam. CT of the abdomen and pelvis revealed a cystic mass in the retrorectal space. MRI was done to further characterize the lesion and this showed a 6.0 × 5.8 cm cystic mass in the retrorectal space extending from the coccyx across midline into the region of the right piriformis and acetabulum and it was deep to the right gluteal musculature (Figure 2). Anoscopy and flexible proctoscopy revealed no disruption of the mucosa or other abnormalities anywhere. Surgical resection was recommended and informed consent was obtained.

**Figure 3:**
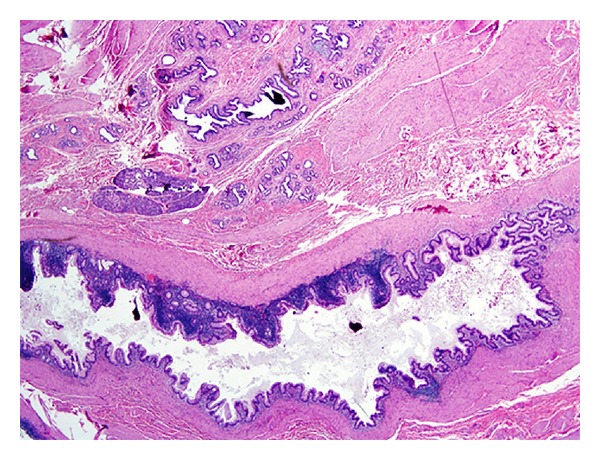
Histologic sections showing pools of mucin associated with an invasive adenocarcinoma and residual teratoma with portion of gut wall and pancreatic parenchyma. (H&E 20x).

The mass was approached operatively with the patient in prone position and through a parasacrococcygeal incision. It was found to be a well-encapsulated cystic lesion and it was completely resected en block with the coccyx. Histologic sections demonstrated an invasive mucinous adenocarcinoma with pools of mucin and moderately to well-differentiated malignant glands coming to within 1 mm of the resected margin. The tumor cells showed immunoreactivity to CDX2, a marker of intestinal differentiation, and coexpression of keratins CK20 and CK7. Smaller areas of teratoma were also identified and consisted of a pancreatic parenchyma, salivary gland tissue, and gut wall. The gut wall was composed of intestinal type mucosa with an underlying layer of smooth muscle resembling muscularis mucosa. These findings are consistent with a teratoma demonstrating endodermal and mesodermal components (Figure 3). Because of these findings and a close surgical margin at the lateral extent of the mass, adjuvant chemoradiation therapy was subsequently recommended.

## 3. Discussion

Tumors that occur in the retrorectal space are challenging because they present with vague symptoms; they are often inaccessible on physical exam, and the anatomic region is not well known to most physicians. Because this “potential space” contains residual totipotent stem cells, a wide variety of neoplasia can develop here and it can present throughout life. There are 2 clinical patterns most commonly observed: (1) neonates presenting with large, predominantly benign tumors that are mature or immature teratomas and (2) infants and children <4 years presenting with teratomas containing malignant cells [10]. In the adult population, teratomas are more rare, but they are also more likely to be malignant due to either recurrence or delay in diagnosis [10]. According to the American Academy of Pediatrics survey by Altman et al. where 400 cases were examined, there was increased rate of malignancy with less apparent lesions and older age at diagnosis: <2 months, 7% girls and 10% boys malignant; >2 months, 48% girls and 67% boys malignant [9].

When sacrococcygeal teratomas are found in the neonatal patient, physical examination and radiologic assessment are used to guide resection. The surgeon must try and avoid nerve injury as bowel and bladder incontinence can occur if damage occurs to the bilateral sacral nerve roots proximal to S3 [4, 11]. Controversy has surrounded the issue of whether or not concomitant coccygectomy should be done along with the resection of these tumors [6, 7, 9, 14–16]. There are multiple studies, which show that nonremoval of the coccyx is a factor for recurrence. For example, Gonzalez-Crussi et al. found that if coccygectomy was not performed, recurrence rates were as high as 37% [17]. Furthermore, the literature has shown that when the lesions do recur, there are increased rates of malignancy [9, 18]. It is notable that in our case, the coccyx was not removed during the original operation and it is possible that if it had been, the recurrence would have been avoided.

These tumors are rare and there is a lack of consensus data for postoperative chemotherapy and radiation. It has been shown that, with early detection and surgical excision, these tumors can be treated with excision and coccygectomy only [17]. If there is malignant transformation within the primary tumor or in a recurrent neoplasm, chemotherapy and radiation should be considered [19]. In this patient's case, there were nests of adenocarcinoma in colonic type tissue, and, in association with its near surgical margin and anatomic location, it was decided to be best treated with radiosensitizing 5-fluorouracil based chemotherapy.

This case is unique because it demonstrates that not only an infant born with a SCT can be seemingly cured with surgery in the neonatal period but also that recurrence can happen decades later. The recurrence can manifest in the anatomic region known as the retrorectal space and the presenting symptoms may be vague. This case also supports the prior findings of increased rates of malignancy in recurrent lesions. Finally, it reinforces the notion that a history of prior resection for SCT in the neonatal period should be noted by physicians caring for the patient even when the index operation was done long ago.
